# A latent profile analysis of Germans’ threat perceptions, intergroup emotions and attitudes toward refugees

**DOI:** 10.1186/s40359-026-04554-x

**Published:** 2026-05-20

**Authors:** Saskia Schubert, Ulrike Kluge, Ursula Hess, Tobias Ringeisen

**Affiliations:** 1 Berlin School of Economics and Law, Alt-Friedrichsfelde 60, Berlin, 10315 Germany; 2 Klinik für Psychiatrie, Charité Universitätsmedizin Berlin, Psychotherapie Bonhoefferweg 3, Berlin, 10117 Germany; 3 Berliner Institut für empirische Integrations- und Migrationsforschung (BIM), Humboldt-Universität zu Berlin, Hannoversche Str. 25, Berlin, 10115 Germany; 4 Institut für Psychologie, Humboldt-Universität zu Berlin, Wolfgang Köhler- Haus, Rudower Chaussee 18, Berlin, 12489 Germany

**Keywords:** Refugees, Attitudes, Intercultural contact, Intergroup emotions, Threat perceptions

## Abstract

**Background:**

Members of receiving societies respond differently to arriving refugees. Person-centered factors can affect the level of perceived threat by refugees, the intensity of intergroup emotions, and attitudes toward refugees. However, it is largely unknown whether members of receiving societies display distinct profiles reflecting the co-occurrence of cognitive-affective-attitudinal responses and person-centered antecedents toward refugees.

**Methods:**

The current study used latent profile analysis to examine distinct response profiles of Germans without migration background (*N* = 910). Participants completed a cross-sectional online survey, covering threat perceptions, intergroup emotions (anxiety, hope, anger, happiness), attitudes toward refugees, and person-centered antecedents (identity as German, intercultural contact, cognitive empathy).

**Results:**

Four latent profiles with distinct cognitive-affective-attitudinal response patterns and antecedents were identified. *The Threatened Angry* (13.1%) displayed highly intense response patterns and low intercultural contact experiences. *The Hopeful Approachable* (28.6%) combined low threat perceptions with more intense positive emotions and attitudes. *The Ambivalent* (41.4%) displayed moderate, yet ambivalent response patterns. *The Ambivalent and Anxious* (16.9%) largely resembled the Ambivalent profile, but reported higher intergroup anxiety and more intercultural contact experiences.

**Conclusion:**

Knowledge about different response patterns may inform the development of intervention programs and information campaigns with group-specific approaches, aiming to potentially lower threat perceptions, raise positive emotions, and address attitudes within this population. In addition, encouraging positive contact experiences between the German majority population and refugees could foster a more differentiated assessment of refugees.

**Supplementary Information:**

The online version contains supplementary material available at 10.1186/s40359-026-04554-x.

Worldwide, migration flows are increasing. Germany hosts the fourth-largest number of refugees worldwide [1]. The arrival and resettlement of refugees, who have fled their home countries to escape violence, conflict, or persecution, can be a stressful process, not only for the refugees themselves but also for members of the receiving societies, as the latter might perceive refugees as foreign and different [2–4]. Thus, perceiving refugees as threatening can be a stressor for members of the majority group [3].

The conditions under which members of the receiving society may perceive refugees as threatening are explained by Integrated Threat Theory (ITT) [5, 6]. This theory defines four different types of threat perceptions that go along with negative affective-attitudinal responses regarding an outgroup: the perception of realistic threat (regarding physical or economic well-being), symbolic threat (regarding one’s own morals, values and norms), negative stereotyping (expected negative characteristics of the outgroup) and intergroup anxiety (feeling of discomfort regarding interactions with the outgroup). The perception of threat is suggested to be triggered by six different antecedents, of which identification with one’s own nationality, as well as intercultural contact experiences and cognitive empathy, the latter in the form of knowledge about the other group, have been shown to be of importance in the context of refugees [7, 8]. This model was later refined, and the present research builds on this refined version, which is described in detail below.

The associations between threat perceptions, their antecedents, resulting intergroup emotions, and attitudes have been well studied in recent years [9–12]. For refugees and members of receiving societies, most studies employed a variable-centered approach, in which unidirectional effects between selected variables were investigated [7, 13–15]. However, even though these models identify average associations across the whole population, they cannot capture intensity patterns of multi-faceted responses that define subgroups of individuals who are expected to react differently to refugees [9, 16]. This, in turn, can lead to the design of interventions that may not only be ineffective but even counterproductive for certain subgroups. For example, where a variable-centered model might suggest a uniform relationship between threat and attitudes, a person-centered approach could reveal some subgroups that differ greatly in their patterns of intergroup emotions but not in the intensity of threat perceptions or attitudes, therefore differentiating emotional response patterns.

These important differences between subgroups would be missed if the study tested only for hypothesized connections between responses for all participants. Conversely, if subgroups exhibit different emotional responses despite similar levels of perceived threat, this would indicate the need for interventions that target the regulation of negative emotions rather than the reduction of threat perceptions per se. If these patterns are not considered, interventions may be developed which focus on the wrong variables - such as emotions or threat perceptions in our example - which could ultimately lead to their failure. Despite these benefits, however, to date very little research has used a person-centered approach to better understand threat perceptions, emotions and attitudes towards refugees and consequently design more effective interventions.

To address this issue, the present research used a person-centered approach based on the assumptions of the refined Integrated Threat Theory with four classes of intergroup emotions [5, 6, 17]. The study aimed to classify Germans into meaningful subgroups through latent profile analysis (LPA), which would allow us to empirically differentiate possible combinations of threat perceptions (symbolic threat, realistic threat, negative stereotypes), the four distinct intergroup emotions (anxiety, hope, anger, and happiness), attitudes towards refugees, as well as person-centered antecedents (identity as German, prior intercultural contact experience, cognitive empathy toward refugees) [18, 19]. Before describing the study in more detail, we will briefly summarize empirical and conceptual findings regarding the antecedents of threat perceptions and attitudes towards refugees.

## Associations of threat perceptions, antecedents and intergroup emotions with attitudes toward refugees

Several models conceptualize intergroup relations and the role of threat perceptions between cultural majorities and migrant or refugee groups [20]. Whereas theories such as the Concordance Model of Acculturation (CMA) [21], Realistic Group Conflict Theory (RGCT) [22], and Symbolic Racism Theory [23] focus on a single type of threat, Integrated Threat Theory (ITT) specifies multiple types of perceived threat [23, 24]. In its original formulation, ITT distinguished between realistic threats (regarding physical or economic well-being) and symbolic threats (regarding one’s own morals, values, and norms) which are conceived of as complementary threat types. Negative stereotyping and intergroup anxiety were later added as further types of threat in the theory [5].

In subsequent interpretations, including those associated with the Intergroup Threat Theory [25], the categorization of threat types has evolved, resulting in conceptual ambiguity. Nonetheless, both frameworks are well supported by empirical research and contribute meaningfully to understanding intergroup dynamics [26]. The present study focused mainly on the ITT, as this model has been validated in numerous studies in the context of migration and refugees [7, 14].

At the same time, to acknowledge the findings of later studies that have applied alternative categorizations of threat types with meaningful empirical results, we adopted an inclusive approach to threat perceptions. Because our research employs a person-centered analytical framework, all threat types outlined in the original ITT were retained to allow for a comprehensive assessment of the complexity of threat perceptions without being constrained by ongoing debates regarding the conceptual status or relative importance of specific threat types. This inclusive approach is specifically suited to a person-centered design, as it allows to observe how these theoretically distinct facets of threat naturally cluster within profiles, rather than pre-defining a singular path of influence.

Moreover, as Riek et al. emphasized in their meta-analysis on intergroup threat and outgroup attitudes, earlier studies demonstrated the value of distinguishing intergroup anxiety from the other three threat types, as intergroup anxiety may reflect an emotional reaction to perceived threat rather than a threat per se. [24, 25] In addition to intergroup anxiety, other intergroup emotions have been conceptually differentiated and empirically examined since [17, 27].

**Fig. 1:**
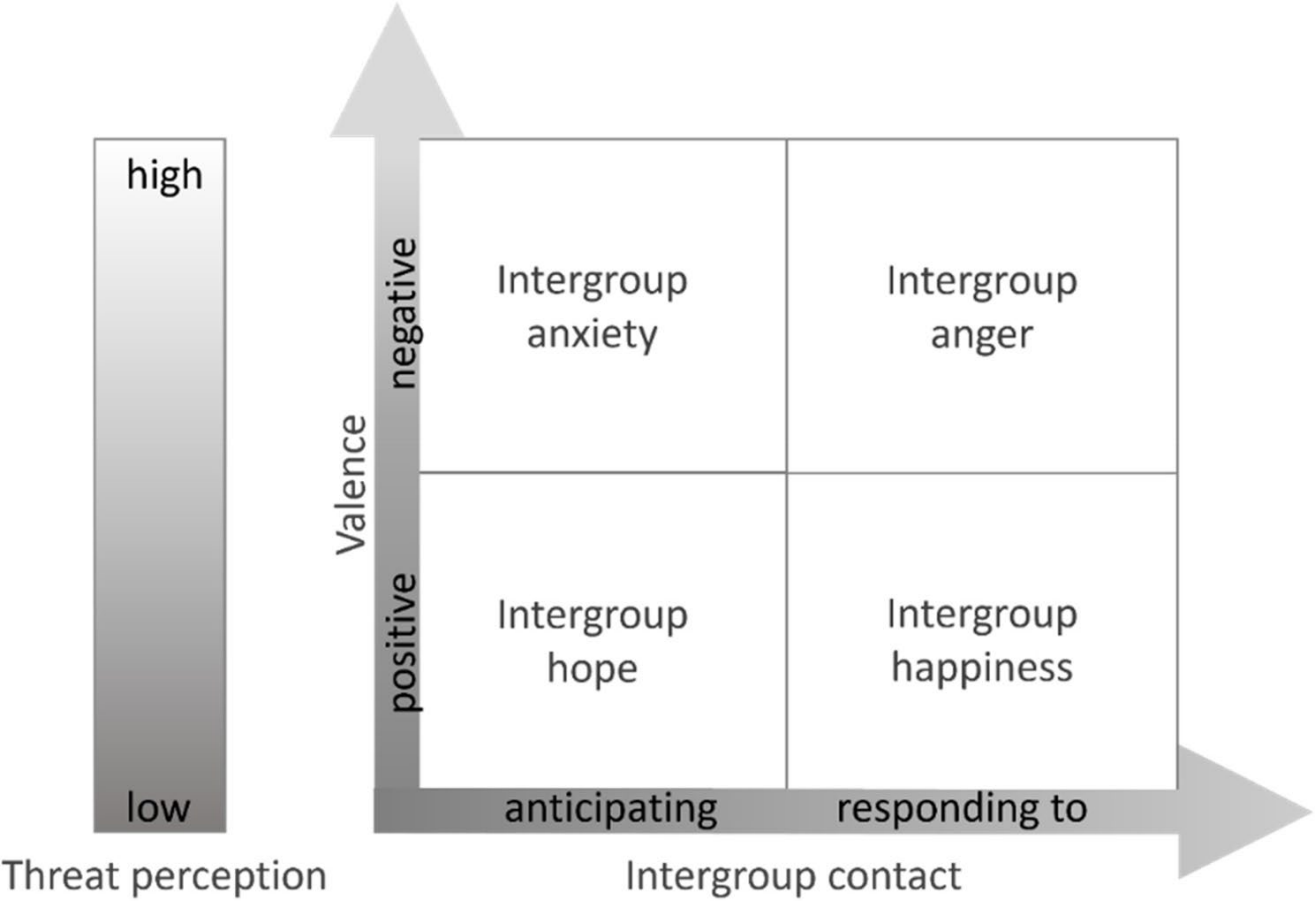
Two-Dimensional Taxonomy of Intergroup Emotions in Intercultural Contexts. Note. Threat perceptions tend to correspond to valence but are conceptually distinct; Own visualization

Intergroup emotions are by definition based on people’s sense of belonging to a certain group and direct intergroup attitudes and behaviors [27, 28]. Their taxonomy can be conceptualized along two dimensions, namely valence^1^ and the level of contact (see Fig. 1) [6, 17, 27]. In intercultural intergroup situations, both positive and negative emotions can occur depending on the valence of the outgroup. As valence reflects a broader evaluative orientation toward an outgroup that is shaped, but not fully determined by perceived threat, higher threat perceptions typically correspond to more negative valence, whereas lower threat perceptions are associated with more positive valence [24]. For example, when Germans (the self-perceived ingroup) perceive refugees arriving in Germany as highly threatening, the model predicts that they experience intergroup anxiety during anticipated contact or intergroup anger in the case of actual contact [27]. However, when threat perceptions are low, positive intergroup emotions, such as intergroup hope and intergroup happiness, should emerge and affect attitudes toward the outgroup positively [17, 27]. Intergroup anxiety and intergroup hope will mainly occur when intergroup interactions are anticipated but have not yet happened, whereas intergroup anger and intergroup happiness emerge in response to actual interactions with a perceived outgroup (see Fig. 1) [6].

**Fig. 2:**
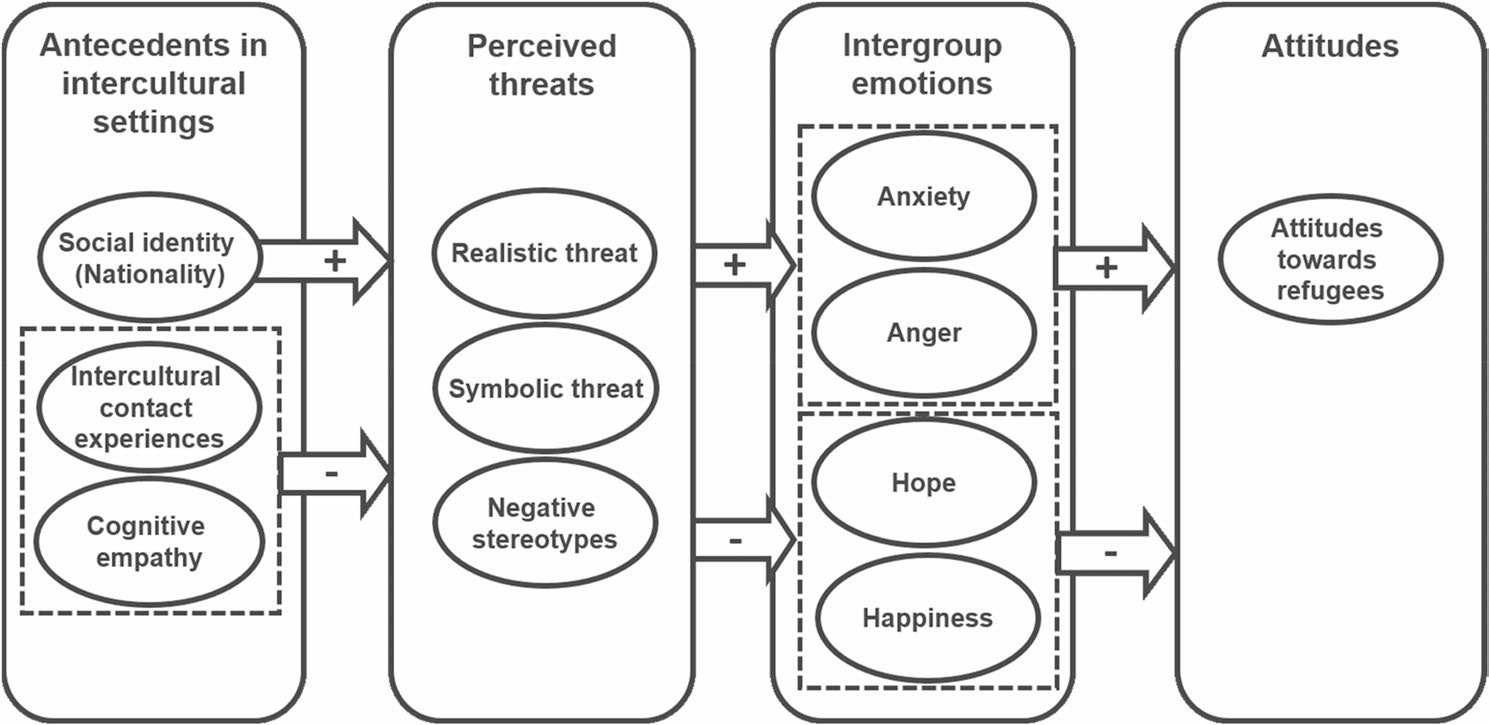
Associations Between Cognitive-Affective-Attitudinal Responses and Antecedents. Note. This figure visualizes associations between threat perceptions, emotions, attitudes, and their antecedents as proposed by Integrated Threat Theory [5, 6, 8]. To enhance readability, some variables are clustered in boxes with dashed lines according to their positive or negative relation with other variables

ITT proposes a sequential chain linking several psychological processes that jointly shape intergroup attitudes. First, antecedent factors provide the contextual basis upon which individuals interpret outgroup-relevant information (see Fig. 2) [5, 29]. In intercultural settings, three person-centered antecedents, namely social identification with one’s own nation, cognitive empathy in the sense of knowledge about the other group’s perspective, and intercultural contact experiences are especially relevant [7, 8]. Second, these antecedents are proposed to influence the emergence of the perceived threats. Third, these threat perceptions should elicit affective responses, which in turn serve as proximal predictors of intergroup attitudes [7, 14, 15].

### Person-centered approach

As noted above, the relationships between threat perceptions, intergroup emotions, and attitudes toward an outgroup in general and, to a lesser extent, toward refugees in particular, have been the subject of several studies that have yielded relevant results and point to practical implications [3, 7]. Yet, so far, there have been relatively few studies using a person-centered approach in the context of the attitudes of receiving societies toward minorities. The existing research includes constructs such as prejudice, motivation to respond without prejudice, right-wing political orientations, and authoritarianism, as well as social dominance orientation [30–34]. Even fewer studies using person-centered approaches included intercultural contact experiences, emotional reactions, social identification with one’s nationality, and different political orientations [9, 30, 35, 36]. Specifically, two studies on attitudes towards Muslim minorities in Germany and the Netherlands found four to five groups that differed in their tolerance of Muslim practices and the strength of positive/negative attitudes toward the minority group [9, 16]. Albada and colleagues found that, of the four groups, the ones with high levels of intercultural contact experiences expressed more positive attitudes. By contrast, symbolic threat perception regarding cultural differences associated with the Islamic faith was more intense in groups with negative attitudes toward this minority group. Other included variables were the social self, the individual and societal discontent. Dangubic et al. examined authoritarianism, status quo conservatism, and unconditional respect. [16] Neither of these studies included participants’ social identification with their own nationality [9, 16]. However, this was done in a study conducted in New Zealand, which also included attitudes toward migrants and cultural diversity, as well as political orientation [35]. The results revealed seven groups that differed regarding their social identification with their own nation, as well as their attitudes toward migrants and political views. The groups that reported the least social identification with their own nation expressed the most positive attitudes toward migrants, while also showing a positive attachment to their own country. In contrast, participants with strong attachment to their country who, simultaneously, displayed moderate identification with their nationality perceived cultural diversity as most threatening to the country and held the most negative attitudes toward migrants.

In Germany, Unzicker and Boehnke identified five profiles that differed in the endorsement of diversity and democracy, as well as the sense of belonging to German society [36]. According to their analysis, the four profiles characterized by a low endorsement of diversity and democracy were associated with a below-average sense of belonging to German society. Of these, the largest group (16.3%) endorsed high levels of anxiety and insecurity.

In sum, research using a person-centered approach has provided interesting and relevant findings regarding clusters of variables that underlie different attitudes toward ethnic outgroups. However, the extant research has focused mainly on attitudes, political orientations, and related concepts. By contrast, in accordance with ITT [5], we were particularly interested in the cognitive-affective-attitudinal responses of Germans as members of the receiving society when thinking about refugees.

### Hypotheses

The present study aims to offer a clearer picture of Germans’ cognitive-affective-attitudinal responses when thinking about refugees. To achieve this, we conducted a large cross-sectional online survey with validated self-report measures. Applying a person-centered approach, we explored whether distinct subgroups of Germans would emerge that differ in their overall profiles, and to determine how many profiles might provide the best representation of our data. Because person-centered methods are designed to reveal whatever naturally occurring configurations best fit the data, we avoided imposing expectations about the exact number or nature of the profiles in advance. Still, based on prior research [9, 16, 30, 36], it seemed plausible to expect groups that would differ in terms of intercultural contact experiences, social identification with being German, and cognitive empathy, as well as their level of positive emotions toward refugees [9, 16].

Based on prior research, it appears plausible that the data may reveal two profiles that differ meaningfully in attitudes toward refugees and in the overall pattern of threat perceptions [9, 16]. Guided by the refined ITT, we expected further that one of the two profiles could potentially be characterized by relatively strong threat perceptions, higher levels of social identification with being German, lower cognitive empathy toward refugees, and a rather low level of intercultural contact experiences. Given the existing literature, it seemed reasonable to anticipate that individuals in such a profile might also experience elevated intergroup anxiety and intergroup anger when thinking about incoming refugees whereas intergroup hope and feelings of intergroup happiness would likely be lower [6, 17]. In turn, this profile could relate to more negative attitudes toward refugees [9, 16].

The second profile was hypothesized to reflect opposite characteristics. Specifically, this group may include individuals who report lower threat perceptions and report substantial knowledge about the emotional concerns of refugees. Furthermore, they would have more intercultural contact experiences and a lower identification with their own nation, as prior research found these antecedents to be negatively associated with threat perception [7, 15, 35, 37]. In terms of intergroup emotions, individuals from this group might report lower levels of anxiety and anger as well as higher levels of hope and happiness [6, 17]. Consequently, it seems reasonable to assume that this profile would be associated with more positive attitudes toward refugees [9, 16].

In addition to the above-mentioned groups, prior studies with a person-centered approach suggested two or more profiles that showed inconsistent patterns regarding threat types, emotional responses, and their attitudes on minorities, ^16,31,^ e.g., by combining moderate-to-slightly-positive attitudes with different intensities of elevated symbolic threat perceptions. ^16^

## Method

### Sample

The sample consisted of 910 Germans (M_age_ = 48.40, SD = 14.79), of whom 460 identified as female (50.5%) and 450 as male (49.5%). Participants had to meet three criteria to be included in the study. They had to (1) have German citizenship, (2) be born in Germany, and (3) have parents who were also born in Germany. The reason for these criteria was that we wanted to examine how people whose intercultural contact experiences had predominantly been acquired outside the family context empathize with refugees. While these restrictive inclusion criteria enhance the internal validity of the study by ensuring a homogeneous sample of the majority society, the limitation of the external validity and generalizability of the findings to the broader, more diverse German population has to be acknowledged.

All participants were at least 18 years old. Three participants did not report completing any level of schooling, 148 had a secondary school qualification (8th grade), 379 completed the secondary school certificate (10th grade), 188 completed A-levels with a higher education entrance qualification, and 192 completed a university or vocational college degree. The average monthly household income of participants was €5980 (income/10,000: M = 0.598, SD = 4.86). The dataset for this study can be freely accessed via an online repository [38].

### Procedures

In early 2016, a large cross-sectional online survey^2^ was developed with a company specializing in internet surveys. The company drew an initial randomized sample of 2,086 people from a large (> 500,000 individuals) sample of the German population that was constructed to be representative with regard to age, gender, education, and place of residence. Of those contacted, 48% responded. Before the start of the survey, participants received a detailed written explanation of the questionnaire procedure and were asked for written informed consent. Participants did not receive compensation, and completion took less than 20 min. Respondents also answered three filter questions to ensure that only Germans meeting the previously mentioned three criteria participated.^3^

### Measures

We used validated self-report measures to capture ten different variables in four variable sections, namely antecedents of threat perception, threat perceptions, intergroup emotions, and attitudes. Except for social identity as German and the intergroup emotions section, a Likert scale from [1] (*I do not agree at all*) to [4] (*I agree completely*) was used (see full list of items in the supplements). To minimize participant fatigue and ensure data quality, we utilized validated short scales or selected items from longer instruments. Selection was based on maintaining a manageable survey length and excluding items with phrasing that might trigger defensive reactions. For all study variables, McDonald’s ω values were ≥ 0.79, of which 7 were > 0.86, which indicates good to excellent reliability (see Table 1).

#### Antecedents of threat perceptions

*Social identity *as German was assessed via a 6-point scale ranging from 1 (does not apply at all) to 6 (applies exactly), that included five statements on attitudes toward social identification as German [39]. An item example is “I am proud to identify with Germany.”

To capture habitual responses to and experiences of *intercultural contact* in general,^4^we used the “enjoyment of intercultural interactions” subscale from the German version of the Intercultural Sensitivity Scale (ISS) [40, 41]. The subscale includes four statements describing how people generally experience and respond to intercultural contact situations. The items are formulated in present tense to capture habitual reactions, providing insight into consistent behaviors, which are based on past experiences (e.g., “I enjoy socializing with people from other cultures”).

*Cognitive empathy* in the sense of adopting refugees’ perspective on their support needs was assessed through a subscale of the Berlin Social Support Scale (BSSS) [42, 43], which consists of three items [8]. The wording was adapted to address the perspective of Germans on refugees (e.g., “When refugees are down, they need someone who boosts their spirits”).

#### Threat perceptions

Based on the refined version of the ITT [5, 6], symbolic threat, realistic threat, and negative stereotyping were included as types of subjective threat perceptions. Realistic threat and symbolic threat were assessed with six items that are used in surveys of xenophobia toward migrants in Germany [44–46]. To capture respondents’ threat perceptions regarding incoming refugees to Germany, all six items were slightly reformulated. Four items assessed perceptions of realistic threat (e.g., “In addition to the foreigners living in Germany, the recently arrived refugees take jobs away from Germans.”) and the two remaining items captured symbolic threats (e.g., “Germany is infiltrated with too many foreign influences due to the numerous refugees.”). Because the two subscales did not show sufficient discriminant validity (see preliminary analysis) symbolic and realistic threat were merged in the following analyses. This aligns with intergroup threat research, which frames both threat types as components of a broader threat construct, while remaining inconclusive about their precise distinction and interrelation [24]. In practice, this means that participants may not clearly discriminate between the two threat types.

Prior research has shown that male refugees are more likely to be discriminated against than female refugees [47], although the majority of refugees in Germany are young men [48]. Based on these findings, we used four items from the German version of the Ambivalence toward Men Inventory to assess negative stereotyping toward male refugees [49, 50]. The wording was slightly adapted to address male refugees in particular. Participants rated four statements such as “When refugees ‘help’ women, they do so only to prove their superiority”.

#### Intergroup emotions

In the current study, participants were given a list of adjectives describing relevant emotions mirroring the procedures described in Kauff et al. [17] We phrased instructions similar to Kauff and colleagues [17]. Specifically, participants were asked to indicate “the extent to which they feel each of the following emotions when they think of the refugees coming to Germany.” The five-point Likert scale ranged from [1] (*not at all)* to [5] (*extremely)*.

*Intergroup anxiety* was assessed using three adjectives proposed by Stephan and Kauff et al., [6, 17] namely anxious, fearful, and worried. For *Intergroup happiness*, we used the adjectives ‘happy’, ‘content’, and ‘relieved’ [17]. To reflect the three components of hope as proposed by Cohen-Chen et al., [51] we used the adjectives ‘eager’ (desire component), ‘confident’ (positive outcome expectation), and ‘hopeful’ (positive feeling regarding the outcome) to capture *Intergroup hope. Intergroup anger*was assessed using the adjectives ‘angry’ and ‘disappointed’ [17, 52].

#### Attitudes

*Positive attitudes*regarding refugees were assessed via a subscale based on the Eurobarometer 53, as validated by Manzoni [53, 54]. As we wanted to concentrate on positive attitudes, we chose the three positively framed statements from the subscale (e.g., “Enjoying the right of asylum in Germany should be easier.”).

### Statistical analyses

To investigate the dimensionality of our instruments, a confirmatory factor analysis was conducted. Model fit was assessed using the parameters recommended by Hu and Bentler [55]. Accordingly, the Chi-Square Test of Model Fit (χ2), the Root Mean Square Error of Approximation (RMSEA) with the 90% confidence intervals, the Comparative Fit Index (CFI), the Tucker-Lewis Index (TLI), and the Standardized Root Mean Square Residuals (SRMR) were examined. The closer the values for the CFI and the TLI are to 1, the better the model’s fit. Model fit values ≥ 0.90 are considered acceptable. For the SRMR and RMSEA however, a value close to 0 is a perfect model fit, while values ≤ 0.06/0.08 can indicate good or acceptable model fit [55]. Since we used manifest variables in latent profile analyses, we computed mean scores from the items of each scale. For correlations between the variables, see Table 1. There was no missing data.

To assess meaningful interindividual differences in cognitive-affective-attitudinal responses and selected antecedents, we conducted latent profile analyses (LPA) [56] to uncover latent groups by determining the most adequate number of profiles across the study variables. For LPA [56], different profile solutions are calculated and compared with an increasing number of defined profiles to then identify the most suitable solution using fit indices. To carry out the LPA, we computed composite continuous mean scores for each variable, which were transformed to a unified rating format from 1 to 4 to harmonize the indicators. This was achieved through linear rescaling, mapping the original scale endpoints (e.g., 1–5 or 1–6) onto the 1–4 metric to ensure comparability while preserving the relative distribution of the scores [56]. This transformation allows for the identification of patterns across diverse constructs; however, it means that the profile scores represent relative intensities within the harmonized 1–4 range rather than absolute values from the original measurement scales. Subsequently, we compared solutions for up to seven profiles. The maximum likelihood estimation with robust standard errors (MLR) was applied to account for non-normality of the data. The fit indices used are Log-likelihood (LL), Akaike Information Criterion (AIC), Bayesian Information Criterion (BIC), Sample-Size-Adjusted BIC (ssaBIC), Mendell-Rubin Likelihood-Ratio-Tests (VLMRT, aLMRT) and Bootstrap LRT.

Regarding the interpretation of AIC, BIC, and ssaBIC, lower values indicate a better model fit when comparing models with different numbers of profiles [56]. However, it is not uncommon for BIC, like other information criteria, to continue decreasing with each class added [49]. Therefore, the decision regarding the best solution should not solely rely on these criteria. LMRT and Bootstrap LRT provide *p*-values that directly compare a model with a defined number of classes to a model with one fewer class. Values ≤ 0.05 indicate significance [56, 57]. All measures are therefore indicators of relative model fit. Another criterion is entropy, where a value > 0.8 indicates an acceptable classification. Finally, none of the profiles in the selected solution should contain fewer than 5% of the sample [58].

To test our profile solution for the significance of expected differences between groups in person-centered antecedents, threat perceptions, intergroup emotions, and attitudes toward refugees, we considered the probabilities for correctly categorizing a person in any given latent profile and conducted analyses of variance (ANOVAs) for each variable using SPSS 27 [59]. For both the confirmatory factor analysis and the subsequent analysis of latent profiles, we used Mplus version 8 [60, 61].

## Results

### Preliminary analysis

To examine the factor structure of our scales, we first conducted a multivariate confirmatory factor analysis. The fit indices reflected an acceptable model fit (model 1, see Table 5 in Appendix). Based on these latent associations, we used the heterotrait–monotrait ratio of correlations (HTMT) to assess the discriminant validity of the study constructs. The HTMT is considered to be more sensitive to violations of discriminant validity than traditional criteria such as the Fornell–Larcker test [62]. HTMT reflects the ratio between (a) the average correlations among indicators that belong to different constructs and (b) the average correlations among indicators measuring the same construct. According to Henseler et al. (2015), HTMT values below 0.85 (strict criterion) or 0.90 (more lenient criterion) indicate that two related constructs can be regarded as sufficiently distinct.

HTMT values for the latent constructs ranged from 0.02 to 0.84, indicating good discriminant validity for nearly all measures. Only the HTMT ratio between symbolic and realistic threat reached 0.92 (latent correlation of 0.94), suggesting that these two constructs did not meet the recommended threshold for adequate discriminant validity. This pattern indicates that participants in our sample experienced both threat types so consistently together that they may be regarded as conceptually indistinguishable. Consequently, we combined the two variables into a single threat subscale. We repeated the confirmatory factor analysis (model 2), which yielded a good model fit (see Table 5 in Appendix). Model 1 and 2 were compared using the CFI difference test. Following Cheung and Rensvold (2002),CFI differences greater than 0.01 suggest meaningful deterioration in model fit, whereas smaller differences are regarded as negligible [63]. Because the observed CFI difference fell below this threshold, we retained the more restrictive CFA (model 2), in which symbolic and realistic threat were represented by a single latent factor. As in model 2 all the standardized factor loadings were satisfactory (> 0.60) [64] and all McDonald’s ω values were good to excellent (≥ 0.79; see Table 1) [65]. We subsequently calculated mean scores for the respective subscales for further analyses.

In terms of latent associations between the study variables and the items’ mean scores, we found several significant associations (see Table 1, latent associations above the diagonal and manifest correlations below the diagonal). Regarding the latter, social identity as German correlated highly positively with both threat perception types (0.337 ≤ *r* ≤.342; *p* <.001), and moderately positively with negative intergroup emotions (0.180 ≤ *r* ≤.191; *p* <.001), while yielding negative associations with the other two antecedents (−0.019 ≤ *r* ≤ -.130; *p* >.05 and *p* <.001), positive intergroup emotions (−0.020 ≤ *r* ≤ -.022; *p* >.05) and with positive attitudes regarding refugees (*r* = -.166; *p* <.001). The negative correlations with cognitive empathy and the two intergroup emotions, hope and happiness, were not significant (−0.014 < *r* < -.022; *p* >.05). Intercultural contact experiences were highly negatively associated with both threat perceptions (−0.447 ≤ *r* ≤ -.546; *p* <.001) and negative intergroup emotions (−0.318 ≤ *r* ≤ -.348; *p* <.001), as well as highly positively correlated with cognitive empathy (*r* =.591; *p* <.001), positive intergroup emotions (0.268 ≤ *r* ≤.460; *p* <.001) and with positive attitudes toward refugees (*r* =.564; *p* <.001). Cognitive empathy toward refugees showed the same pattern of intercorrelations as intercultural contact experiences, except for slightly stronger positive correlations with intergroup happiness (*r* =.425; *p* <.001) and positive attitudes toward refugees (*r* =.607; *p* <.001). Symbolic-realistic threat and stereotyping correlated highly positively (*r* =.760; *p* <.001). Further, positive intergroup emotions were positively correlated with each other (*r* =.739; *p* <.001) and with positive attitudes regarding refugees (0.425 ≤ *r* ≤.569; *p* <.001), whereas negative emotions were positively associated with each (*r* =.692; *p* <.001) other and negatively with attitudes toward refugees (−0.357 ≤ *r* ≤ -.424; *p* <.001).

**Table 1:** Latent and manifest correlations of perceived threats, their antecedents, attitudes and intergroup emotions

Constructs	M	SD	1.	2.	3.	4.	5.	6.	7.	8.	9.	10.
1. Social identity as German	3.151	0.591	0.937	− 0.136*	− 0.022	0.360**	0.363**	0.213**	0.207**	− 0.014	− 0.014	− 0.173**
2. Intercultural contact experiences	2.942	0.692	− 0.130**	0.918	0.642**	− 0.571**	− 0.486**	− 0.363**	− 0.416**	0.289**	0.516**	0.667**
3. Cognitive empathy			− 0.019	0.591**	0.898	− 0.590**	− 0.511**	− 0.365**	− 0.513**	0.247**	0.683**	0.720**
4. Symbolic/realistic threat	2.381	0.819	0.337**	− 0.546**	− 0.548**	0.897	0.812**	0.656**	0.713**	− 0.287**	− 0.548**	− 0.679**
5. Negative stereotyping	2.413	0.836	0.342**	− 0.447**	− 0.465**	0.760**	0.916	0.610**	0.656**	− 0.199**	− 0.437**	− 0.550**
6. Intergroup anxiety	2.276	0.720	0.191**	− 0.318**	− 0.311**	0.508**	0.529**	0.823	0.860**	− 0.225**	− 0.428**	− 0.429**
7. Intergroup anger	2.177	0.854	0.180**	− 0.348**	− 0.442**	0.618**	0.554**	0.692**	0.792	− 0.226**	− 0.483**	− 0.553**
8. Intergroup happiness	1.916	0.665	− 0.020	0.268**	0.425**	− 0.225**	− 0.169	− 0.192**	− 0.148**	0.872	0.818**	0.481**
9. Intergroup hope	2.230	0.702	− 0.022	0.460**	0.447**	− 0.460**	− 0.370	− 0.345**	− 0.349**	0.739**	0.858	0.683**
10. Positive attitudes	2.339	0.646	− 0.166**	0.564**	0.607**	− 0.574**	− 0.465**	− 0.357**	− 0.424**	0.425**	0.569**	0.793

Note. *N* = 910. **p<.001; *p<.05 (two-tailed); Numbers above the diagonal depict latent correlations, while the numbers below the diagonal depict manifest correlations; the diagonal depicts McDonald’s ω as an estimate of reliability coefficients

Table 2presents model fit information for the latent profile analyses for solutions ranging from one to seven profiles. Entropy values were high for all estimated models, and except for VLMRT and aLMRT, fit indices continued to improve with the addition of latent profiles. Regarding VLMRT and aLMRT, a four-profile solution was favorable, as there was no significant improvement with the addition of a fifth profile. The four-profile solution is consistent with prior research and represents conceptually meaningful combinations of threat perceptions, intergroup emotions, and attitudes toward refugees [37]. Moreover, models with more than four profiles did not reveal additional subgroups that were meaningfully distinct from the first four. Further, the six-profile-solution as well as the seven-profile-solution contained one profile with fewer than 5% of classified individuals. Overall, this suggests that a four-profile-solution is the most appropriate [16, 57]. These findings were confirmed in a second set of analyses with different start values. The high entropy (Table 2) and average latent profile probabilities (Table 3) support the stability of this solution and indicate that the assumption of local independence was sufficiently met, particularly after merging the threat indicators.

**Table 2:** Fit indices of the Latent Profile Analyses

Fit indices	1	2	3	4	5	6	7
Cell frequencies per class (%)							
1	910 (100)	472 (51.87)	270 (29.67)	119 (13.08)	245 (26.92)	63 (6.92)	66 (7.25)
2		438 (48.13)	440 (48.35)	379 (41.65)	71 (7.08)	234 (25.71)	105 (11.54)
3			200 (21.98)	259 (28.46)	342 (37.58)	292 (32.09)	170 (18.68)
4				153 (16.81)	160 (17.58)	106 (11.65)	196 (21.54)
5					92 (10.11)	198 (21.76)	78 (8.57)
6						17 (1.87)	278 (30.55)
7							17 (1.87)
Model fit information							
No. of free parameters	32	49	66	83	100	117	134
LL	−18094.223	−16277.623	−15441.808	−15103.940	−14808.590	−14524.974	−14313.321
AIC	36252.446	32653.247	31015.616	30373.879	29817.179	29283.948	28894.642
BIC	36406.477	32889.106	31333.304	30773.395	30298.524	29847.121	29539.644
ssaBIC	36304.849	32733.489	31123.697	30509.799	29980.938	29475.546	29114.079
Diminishing returns		1–2	2–3	3–4	4–5	5–6	6–7
Diff. AIC		3599.199	1637.631	641.737	556.700	533.231	389.306
Diff. BIC		3517.371	1555.802	559.909	474.871	451.403	307.477
Diff. ssaBIC		3571.360	1609.792	613.898	528.861	505.392	361.487
Entropy		0.880	0.924	0.914	0.918	0.932	0.933
VLMRT		0.0007	< 0.0001	0.0097	0.6565	0.2784	0.4369
aLMRT		0.0007	< 0.0001	0.0101	0.6577	0.2796	0.4377
PBLRT		< 0.0001	< 0.0001	< 0.0001	< 0.0001	< 0.0001	< 0.0001

Note. Log-Likelihood, *AIC* Akaike‘s Information Criterion, *BIC* Bayesian Information Criterion, *ssaBIC* Sample-size adjusted Bayesian Information Criterion, *VLMRT* Vuong-Lo-Mendell-Rubin Likelihood-Ratio Test (*p*-value), *aLMRT* Lo-Mendell-Rubin adjusted Likelihood-Ratio Test (*p*-value), *PBLRT* Parametric Bootstrap-Likelihood-Ratio Test (*p*-value)

Average profile probabilities varied between 0.921 and 0.975, indicating a high probability of correct categorization of the individuals into the different profiles (see Table 3).

**Table 3:** Average Latent Profile Probabilities for Most Likely Latent Profile Membership (Row) by Latent Profile (Column)

Profile	Name	1	2	3	4
1	The Threatened Angry	0.963	0.012	0.000	0.025
2	The Ambivalent	0.003	0.950	0.021	0.026
3	The Hopeful Approachable	0.000	0.025	0.975	0.000
4	The Ambivalent and Anxious	0.022	0.057	0.000	0.921

Note. Values along the diagonal represent the average probability that a person in a given latent profile was correctly categorized as belonging to that profile

In the following, the four profiles are described in more detail. Differences across profiles for each scale were examined conducting analyses of variance (ANOVAs) (see Table 4) [66].

**Table 4:** Descriptive comparison of latent profiles across study variables (Welch’s ANOVA and Games-Howell post-hoc tests) and demographic variables

	Profiles				
	1 The Threatened Angry	2 The Ambivalent	3 The Hopeful Approachable	4 The Ambivalent and Anxious	Significant profile differences**
N (%)	119 (13.1%)	379 (41.4%)	259 (28.6%)	153 (16.9%)	

**Variables**	**M ± SD**	**M ± SD**	**M ± SD**	**M ± SD**	
Social Identity	3.40 ± 0.76	3.18 ± 0.72	2.90 ± 0.84	3.30 ± 0.64	1, 4, 2 > 3
Intercultural contact experiences	1.97 ± 0.78	2.75 ± 0.73	3.57 ± 0.52	3.10 ± 0.61	3 > 4 > 2 > 1
Cognitive empathy	1.86 ± 0.75	2.83 ± 0.67	3.56 ± 0.47	3.27 ± 0.57	3 > 4 > 2 > 1
Symbolic/realistic threat	3.55 ± 0.40	2.50 ± 0.67	1.43 ± 0.51	2.77 ± 0.66	1 > 4 > 2 > 3
Negative stereotypes	3.44 ± 0.60	2.53 ± 0.73	1.54 ± 0.59	2.77 ± 0.72	1 > 4 > 2 > 3
Intergroup anxiety	3.35 ± 0.58	2.16 ± 0.39	1.38 ± 0.40	3.24 ± 0.41	1, 4 > 2 > 3
Intergroup hope	1.21 ± 0.39	2.24 ± 0.65	2.72 ± 0.78	2.17 ± 0.88	3 > 2, 4 > 1
Intergroup happiness	1.15 ± 0.35	2.02 ± 0.69	2.10 ± 0.87	1.94 ± 0.93	3, 2, 4 > 1
Intergroup anger	3.42 ± 0.63	2.15 ± 0.65	1.30 ± 0.47	2.75 ± 0.73	1 > 4 > 2 > 3
Positive attitudes	1.34 ± 0.41	2.17 ± 0.58	3.00 ± 0.64	2.41 ± 0.77	3 > 4 > 2 > 1
Demographic variables					
Age	49.86	47.77	49.26	47.36	
Share of males in %	36.1	53.6	56	38.6	
Education beyond secondary school in %	21	38.3	55.2	43.8	

	**df**	**F**	***p*** **-value**	**partial η2**	
Social identity	3	16.659	< 0.001	0.052	
Intercultural contact experiences	3	176.308	< 0.001	0.369	
Cognitive empathy	3	227.485	< 0.001	0.430	
Symbolic & realistic threat	3	397.347	< 0.001	0.568	
Negative stereotypes	3	254.705	< 0.001	0.458	
Intergroup anxiety	3	909.674	< 0.001	0.751	
Intergroup anger	3	382.116	< 0.001	0.559	
Intergroup hope	3	123.397	< 0.001	0.290	
Intergroup happiness	3	47.803	< 0.001	0.137	
Positive attitudes	3	211.276	< 0.001	0.412	
Age	3	1.108	0.345	0.004	
Gender	3	7.721	< 0.001	0.025	
Education	3	14.166	< 0.001	0.045	

Note: Welsh’s ANOVA, Games-Howell post-hoc test

** All pairwise differences were significant at *p* <.01; effect sizes were generally large (see ηp2)

**Fig. 3:**
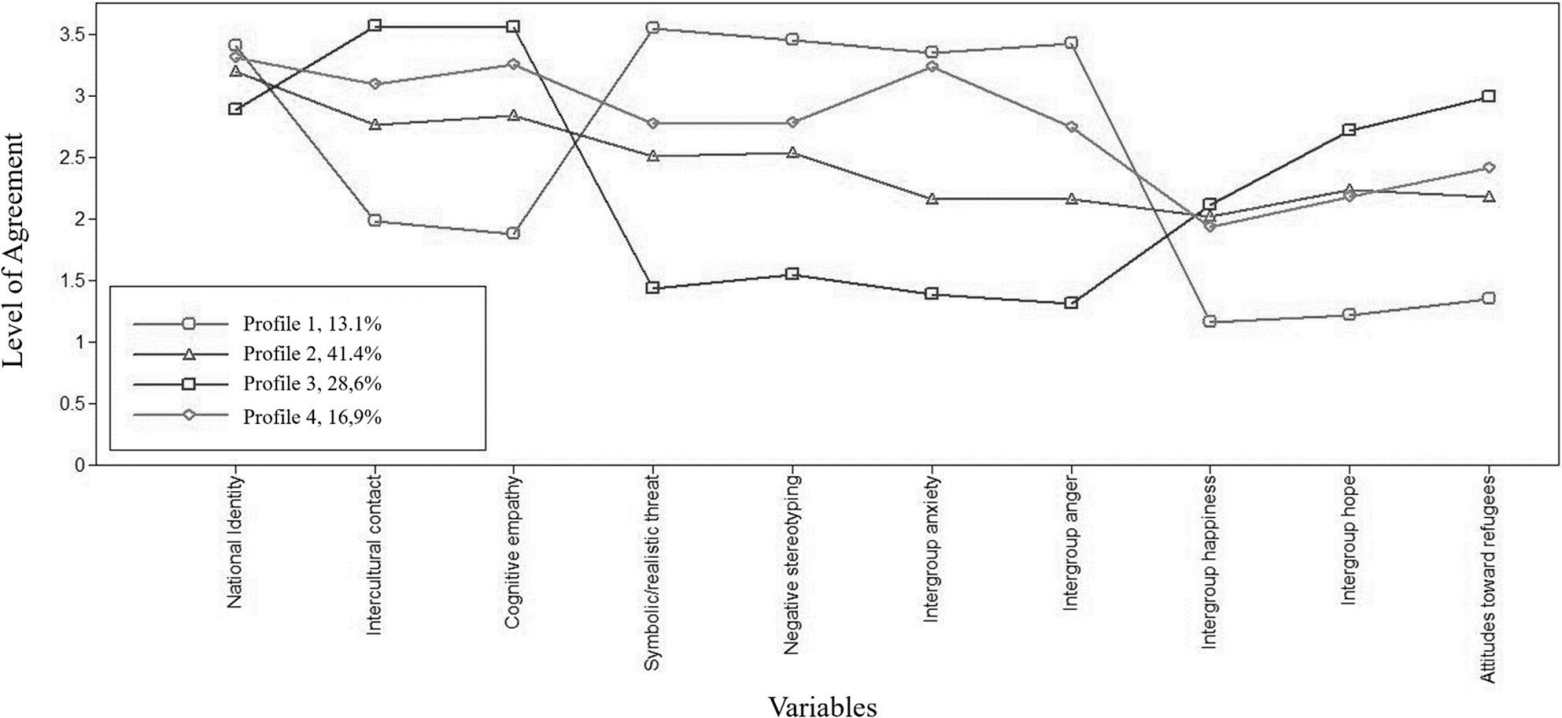
Four-Profile Solution depicting the Ten Study Variables. *Note.* Order of variables: 1 = Social identity as German, 2 = Intercultural contact experiences, 3 = Symbolic and realistic threat, 4 = Negative stereotypes, 5 = Intergroup anxiety, 6 = Intergroup anger, 7 = Intergroup happiness, 8 = Intergroup hope, 9 = Cognitive empathy, 10 = Positive attitudes on refugees; Transformed composite with decimal mean scores ranged from 1 (I *don’t agree at all)* to 4 (I *agree completely*)

Figure 3 displays the mean scores for the perceived threat from refugees, the participants’ person-centered antecedents, attitudes toward refugees, as well as related emotions for the four profiles. The profiles did not differ significantly for age and showed only slight differences in gender distribution and educational levels (see Table 4).

The first profile (13.1% of the participants) is characterized by low intercultural contact experiences, a very high intensity of threat perceptions, as well as strong intergroup anxiety and anger, accompanied by very low intergroup happiness and hope with strongly negative attitudes regarding refugees and moderately low cognitive empathy toward refugees. Participants in this profile felt by far the most threatened by refugees and experienced the most negative intergroup emotions, which is why we labeled this profile *the Threatened Angry*.

Representing an intermediate position, the second profile consists of the largest subgroup (41.4% of the participants) and can be characterized by moderately strong threat perceptions accompanied by moderately high levels of intercultural contact experiences as well as moderately strong positive and negative intergroup emotions. Attitudes toward refugees were moderately positive and cognitive empathy was elevated. We labeled this profile *the Ambivalent.*

Representing the opposite pole, the third profile (28.6% of the participants) reported a high level of prior intercultural contact experiences, a relatively low level of social identification with being German (compared to the other profiles) and very low threat perceptions as well as low anxiety and anger. Further, participants expressed moderately strong intergroup happiness, strong intergroup hope and generally positive attitudes toward refugees. Their level of cognitive empathy was very high as well. As this profile displays a generally positive demeanor toward refugees, we labeled it *the Hopeful Approachable*.

The fourth profile (16.9% of participants) features a relatively high level of intercultural contact experiences and a high level of cognitive empathy alongside threat perceptions, intergroup anxiety, and elevated anger. Yet at the same time, participants reported moderate intergroup happiness and hope, and generally positive attitudes towards refugees. Albeit mostly similar to the second profile, the spike in intergroup anxiety is an important distinction. To emphasize both the similarities in patterns and the main difference from the second profile, we labeled this profile the *Ambivalent and Anxious.*

To characterize the identified profiles, we conducted Welch’s analyses of variance (ANOVAs) and the Games-Howell post hoc test. The results show that all four groups differed significantly regarding the ten variables we included in the LPA (see Table 4). All pairwise differences were significant at *p* <.01. Most effect sizes were large (ranging from η_p_^2^ = 0.14 to η_p_^2^ = 0.75); the effect size for social identity as German was relatively smaller (η_p_^2^ = 0.05), but still corresponded to d = 0.46.

Comparing the groups, *the Threatened Angry* showed the strongest social identity, whereas *the Hopeful Approachable* showed the weakest. Generally, social identity as a German only differed slightly between the profiles (see Table 4). Regarding intercultural contact experiences, *the Threatened Angry* displayed by far the lowest level of intercultural contact experiences compared to the other groups, of which two reported a relatively high rate of the experiences. The *Hopeful Approachable* reported the most intercultural contact experiences.

Compared to the other groups, *the Threatened Angry* indicated the highest level of perceived threat whereas the Hopeful Approachable reported the lowest. *The Ambivalent* and *the Ambivalent and Anxious* both indicated moderate positive and negative emotional responses. Regarding intergroup emotions, *the Threatened Angry* reported the strongest negative intergroup emotions, while intergroup hope and happiness were very low. Generally, the remaining groups showed more positive intergroup emotions, while their differences in intergroup happiness were relatively small. However, they differed significantly regarding their level of negative emotions toward refugees, with the *Ambivalent and Anxious* displaying intergroup anxiety almost as strongly as *the Threatened Angry*.

In contrast to that similarity, when comparing the level of cognitive empathy toward refugees, *the Ambivalent and Anxious* reported the same high level as *the Hopeful Approachable*, followed by *the Ambivalent* whereas *the Threatened Angry* reported relatively low levels. In light of the above-described profiles, it follows that *the Threatened Angry* hold the least positive attitudes toward refugees and their rights, with the other groups reporting moderately to clearly positive attitudes.

Generally, the biggest differences between the profiles were found for perceived threat and negative intergroup emotions as well as intercultural contact experiences. In contrast, positive intergroup emotions, as well as social identity and cognitive empathy differed less. The differences between the groups regarding their attitudes were also moderate. This asymmetry suggests that the profiles are primarily distinguished by their varying levels of perceived hostility and threat, while positive affective responses remain more uniform across the ambivalent and positive subgroups.

Beyond psychological variables, the profiles also showed a clear demographic gradient. Specifically, higher education levels were reported for the profiles with more positive and ambivalent attitudes. Interestingly, while the *Threatened Angry* were characterized by the lowest education level and a predominantly female composition (63.9%), the *Ambivalent* and the *Hopeful Approachable* showed a balanced and a slightly male-dominated gender distribution, respectively.

Considering the characteristics of the *Threatened Angry* and the *Hopeful Approachable*, our hypotheses regarding two opposing profiles in terms of levels of cognitive empathy, threat perceptions, attitudes, and emotions towards refugees, as well as of their social identification with being German and intercultural contact experiences can be accepted. Our finding of two additional profiles also aligns with the findings in prior studies.

## Discussion

We aimed to investigate the cognitive-affective-attitudinal responses of Germans when thinking of refugees with the goal to identify subgroups that differ in threat perceptions, relevant antecedents and emotional responses, as well as their attitudes toward refugees. Using latent profile analyses, we identified four subgroups among the German participants, which showed differentiated individual combinations of variables.

### Interpretation of profile-specific findings

Before discussing the four profiles, it is important to note that the interpretations offered below focus on the patterns emerging from the observed variables. Where we draw on psychological mechanisms that were not directly measured (e.g., general social anxiety or perceptions of societal control loss), these explanations are intended as theoretically informed rather than direct empirical conclusions and should be understood as speculative.

The *Hopeful Approachable* reported a very low sense of threat combined with the most positive intercultural contact experiences and high cognitive empathy toward refugees. Their attitudes toward refugees were positive, and intergroup hope was pronounced. However, compared to intergroup hope, intergroup happiness was quite low. One reason may be that the participants, even though they reported high levels of experiences with people from different cultures, nonetheless had little to no contact with the specific group of refugees in their everyday life. As intergroup happiness is linked to a contact that had already taken place, it would make sense that happiness was lower than hope, which is an anticipatory reaction (see Fig. 1). Another reason may be that participants who belong to this profile were hopeful toward refugees arriving in Germany but aware of the challenges that intercultural interaction situations hold for everyone included, which may diminish intergroup happiness.

In contrast to *the Hopeful Approachable*, *the Threatened Angry* were characterized by a high sense of threat, strong intergroup anxiety and anger as well as negative attitudes toward refugees. As expected, participants with this profile reported few intercultural contact experiences. However, their cognitive empathy regarding refugees was not below average, but rather moderate. This suggests that although members of this group appear to have some cognitive understanding of refugees’ emotional needs, threat perceptions and negative emotional responses may be more influential. Because our study did not assess the specific mechanisms by which threat might outweigh empathic tendencies, this interpretation should be viewed as a theoretical inference rather than a definitive conclusion.

*The Ambivalent* were our largest group. They showed moderately positive attitudes and threat perceptions. Positive as well as negative emotional responses were on an average level. However, knowledge about the emotional needs of refugees was clearly greater, which may indicate that the largest group of the German participants relies more on fact-based assessments of the arrival refugees’ situation in Germany. As information processing was not directly assessed, this interpretation remains tentative.

In contrast to *the Ambivalent*, *the Ambivalent and Anxious *reported strong negative intergroup emotions and were characterized by a strong sense of being threatened, yet, at the same time, reported positive attitudes toward refugees. Their awareness of the emotional concerns of refugees combined with very strong intergroup anxiety is a rather unexpected combination, which makes this profile the most interesting. Prior studies do not suggest that the cognitive level of empathy might be counteracted by anxiety and threat perception [9, 16, 28]. This group reported an elevated level of general intercultural contact experiences, which distinguishes them from *the Threatened Angry*, despite sharing a high level of intergroup anxiety. The combination of variables in *the Ambivalent and Anxious *profile may indicate that the presence of intercultural contact experiences is associated with positive attitudes, even when threat perception and interactional anxiety are pronounced [13]. Another possible explanation for the variable combination could be that people in this group are generally more socially anxious, which would make them more prone to feeling anxious and threatened by intergroup interactions [6].

### Theoretical Integration and contextualization

The composition of the four different profiles did not differ significantly regarding age. Gender and the level of education only showed slight differences. Specifically, the *Hopeful Approachable* were the most highly educated and slightly more male-dominated, whereas the *Threatened Angry* were predominantly female and reported the lowest education. Albeit speculative, this pattern may suggest that higher education may serve as a resource for mitigating threat perceptions, as previous research has linked education to lower perceived intergroup threat and more positive attitudes toward outgroups [5, 9, 25]. Furthermore, the predominantly female composition of the *Threatened Angry *could tentatively align with some studies suggesting that women may report stronger intergroup threat perceptions when threats are framed in terms of social stability or personal safety [6, 29]. However, as these specific perceptions were not directly measured, this interpretation remains a theoretically informed hypothesis.

Interestingly, the identification with being German did not differ strongly across the profiles. Although positive intergroup emotions, particularly happiness, showed little variation between the profiles and remained at a relatively low level, the values for negative intergroup emotions, diverged significantly between the profiles. This emotional asymmetry may suggest that the differentiation between the profiles is driven mainly by the presence or absence of negative affect rather than by varying degrees of positive emotions [27]. The observed levels of negative intergroup emotions mostly correspond to the expected levels of cognitive and attitudinal variables, which differ greatly between the profiles. However, our results offer an encouraging picture of societal cohesion, as the majority of respondents reported low levels of negative intergroup emotions (54.5%), generally positive attitudes toward refugees (86.9%) and a rather low sense of threat (70%). These numbers are higher than those reported in previous studies (61.1% in Unzicker & Boehnke [34], 51.7% in Albada et al. [9]). We found only one relatively small group that showed highly positive emotions and attitudes toward refugees, as well as one small group that reported predominantly negative intergroup emotions and attitudes toward refugees, findings that are in line with prior studies [9, 16, 67]. By contrast, the largest group revealed mainly moderate views regarding refugees, similar to the study by Unzicker and Boehnke [34]. This suggests a balanced view of refugees that combines both some negative and some positive elements with no extremes. To our surprise, one profile included positive attitudes toward refugees but at the same time negative intergroup emotions and threat perceptions. This is a new finding that has not been reported yet. Advances in intergroup emotion research emphasize that attitudes toward outgroups are often shaped by complex constellations of group-based emotions rather than single affective responses [27, 28]. From this perspective, the coexistence of positive attitudes toward refugees alongside elevated intergroup anxiety and threat perceptions observed in the present study may reflect the simultaneous activation of different emotional appraisals in intergroup contexts [68]. The elevated threat perceptions and negative intergroup emotions could possibly be rooted in experiencing a sense of strangerhood and a subsequent misattribution of these anxieties onto refugees. The participants in this group reported a high level of intercultural contact experiences and cognitive empathy towards refugees. Albeit speculative, this pattern may reflect a nuanced differentiation between general attitudes toward refugees and emotional reactions in specific intergroup situations. The participants may succeed in reflecting more on the differentiated reasons for their negative perceptions, thus still holding positive attitudes toward refugees. This explanation would contribute to the previous research by Landman et al., who identified a combination of threat perceptions and a lack of compassion, a facet of empathy, to be associated with negative attitudes towards refugees. [69] However, the reported threat type, described as altruistic threat to refugees’ care was not included in this study, limiting the comparability of the findings. Even though speculative, another possible explanation for the high level of reported intergroup anxiety and threat perceptions could lie in contextual factors, such as perceptions of control loss regarding societal processes in relation to forced migration or a loss of trust in the governmental abilities to handle the influx of refugees. However, additional variables may be needed to better understand the unexpected pattern, which, at first glance, does not seem psychologically coherent.

### Implications

This study provides important insights into intergroup threat and intergroup contact theories. The findings highlight the complexity of threat perceptions, particularly how intergroup anxiety operates independently of symbolic and realistic threats which requires targeted interventions. Cognitive empathy emerged as a key component associated with positive attitudes, even in the presence of heightened threat perceptions, suggesting its potential as a moderating factor in threat models.

The findings of this study have important implications for designing interventions aimed at reducing threat perceptions, mitigating negative intergroup emotions, and fostering positive attitudes toward refugees. Tailored interventions are essential, given the distinct cognitive-affective-attitudinal profiles identified in this study. Although the proposed interventions are grounded in established theoretical frameworks and the patterns observed in our data, some underlying psychological mechanisms discussed above were not directly measured. Therefore, the recommendations should be interpreted as theoretically informed but not empirically validated within the present study. The *Threatened Angry* exhibited heightened threat perceptions, intense negative intergroup emotions such as anger and anxiety, and the least positive attitudes toward refugees. Interventions for this group should therefore prioritize reductions in perceived threats and facilitating cognitive empathy. Grounded in the contact hypothesis, reducing these negative emotions is a prerequisite for any successful interaction [70]. First, empathy-focused campaigns featuring personal narratives of refugees could humanize their experiences and address prevalent stereotypes [71]. Additionally, moderated community forums can provide a safe space for individuals to articulate their concerns while receiving evidence-based information that counters negative stereotypes [71]. Structured, incremental contact programs - beginning with indirect exposure through media and progressing to in-person interactions in low-stress environments - may further reduce intergroup anxiety. Finally, media literacy initiatives can empower individuals to critically assess sensationalized portrayals of refugees, which often exacerbate fears.

The *Ambivalent*, characterized by moderate threat perceptions and a balanced mix of positive and negative emotions, may benefit from interventions that deepen understanding of refugees and reinforce positive intergroup emotions. This aligns with the ITT, stating that increasing outgroup knowledge can stabilize moderate attitudes by reducing symbolic threat [5]. Fact-based campaigns highlighting the contributions of refugees to German society (economically, culturally, and socially) could help mitigate remaining concerns. Interactive educational sessions that explore refugees’ cultural backgrounds and personal stories may further foster a sense of shared humanity. Moreover, highlighting success stories of integration through documentaries and media campaigns could further enhance intergroup hope. Encouraging members of this group to participate in volunteer programs, such as language tutoring or cultural exchange activities, could provide first-hand exposure to the positive outcomes of refugee integration [72].

The *Hopeful Approachable *displayed the most positive attitudes and emotions toward refugees, along with low threat perceptions and high levels of intercultural contact experiences. For this group, interventions should focus on maintaining and amplifying their supportive attitudes while fostering intergroup happiness. Direct engagement opportunities, such as mentorship or buddy programs, could strengthen their positive contributions by facilitating meaningful, one-on-one interactions with refugees [5, 73]. Community-building activities, including cultural festivals or sports events, offer avenues for fostering inclusive ingroups and celebrating diversity. Empowering this group to act as advocates for refugee integration through training, resources, and platforms to share their experiences, may amplify their influence and inspire positive attitudes in other groups. Additionally, these individuals could benefit from training to address systemic barriers faced by refugees, thereby promoting structural changes in addition to interpersonal connections.

The *Ambivalent and Anxious* exhibited relatively high levels of intercultural contact experiences and cognitive empathy but reported pronounced intergroup anxiety and threat perceptions. This unique combination suggests that interventions for this group could focus on reducing intergroup anxiety and fostering emotional resilience to prevent negative emotional responses from interfering with their cognitive empathy [6]. Intercultural communication training could equip this group with practical strategies for navigating intergroup interactions, thereby addressing feelings of discomfort [73]. Structured, low-pressure contact programs, such as cultural workshops or guided dialogues, may help mitigate anxiety by promoting positive, controlled interactions with refugees. Normalizing intergroup anxiety through psychoeducational resources—framing it as a natural response that can be managed—could also prove beneficial [74]. Finally, role-model exposure, where individuals who initially experienced similar anxieties share their journeys toward positive intergroup relationships, may inspire hope and demonstrate pathways for overcoming these challenges.

Across all four profiles, a notable finding was the relatively low level of reported intergroup happiness. In addition to the factors already discussed, broader societal dynamics may also play a role. Perceptions of forced migration are driven by the interplay of perceived threats and humanitarian considerations [71]. The low levels of intergroup happiness may thus reflect the heated political and public debate surrounding migration and refugees in Germany, as the complex and contested nature of this topic might be less strongly associated with positive emotions, regardless of individuals’ personal stances. This underscores the importance of fostering empathy (e.g., through campaigns featuring humanitarian concerns) [71] and supporting positive contact experiences with refugees, as prior research has demonstrated the central role of positive interactions in reducing prejudice and improving intergroup attitudes [75]. Programs that facilitate inclusive group activities, such as sports or shared community projects, can create opportunities for positive intergroup experiences, reduce intergroup anger, and enhance both hope and happiness [5, 17, 70].

Overall, these tailored interventions emphasize the importance of addressing the specific emotional and cognitive profiles of receiving society members. Such differentiated approaches have the potential to foster more inclusive attitudes, reduce perceived threats, and promote harmonious coexistence between refugees and host communities. Future research should evaluate the effectiveness of these interventions across diverse social and cultural contexts.

### Strengths and limitations

Our study has a number of strengths. To our knowledge, it is among the first studies to include all relevant variables of the Integrated Threat Theory and to identify different profiles among them [5]. With its focus on cognitive-affective responses to threat perceptions, rather than the political orientation of the participants, it offers a new perspective and a basis for future research, as well as more effective designs of interventions or informational campaigns.

Some limitations of our results must nonetheless be taken into account. The study focused on the perspective of Germans toward refugees as perceived outgroup, emphasizing general patterns rather than variability within the outgroup or differences between specific refugee groups. Conducting comparative analyses would be a valuable next step.

The present study focused on Germans who gathered intercultural experiences mainly outside their family context. We included participants who were born in Germany and whose parents were born in Germany. However, we did not exclude Germans whose grandparents migrated to Germany. Recording the birthplaces of grandparents has proven difficult, as respondents often omit this information. Furthermore, the relevance of the migration history of the grandparents’ generation for the contact experiences related to their corresponding cultural background(s) has not yet been scientifically determined [76]. By focusing on non-migrant Germans with two German-born parents, we prioritized internal validity to isolate majority-society patterns, which necessarily limits the external validity of the findings. Although our sample focused on ethnic Germans born in Germany with German-born parents, the identified profiles may reflect general patterns of intergroup emotions and threat perceptions relevant to younger Germans, individuals with mixed heritage, or broader European contexts. However, the specific content and salience of perceived threats may vary across groups, and further research is needed to assess the generalizability of these profiles.

Further, following the contact hypothesis, the study focused on positive intercultural contact, although it is possible that negative or neutral contact experiences also may an influence on profile assignments [26]. Given the large number of variables already included in the present study, we had to limit the scope of contact measures. Nevertheless, future research should consider incorporating negative contact experiences, as suggested by Croucher [26], to provide a more comprehensive understanding of cognitive-affective-attitudinal responses.

In contrast to variable-based analysis, the person-centered approach can provide a more differentiated picture of the German participants. However, it should be noted that the exclusive reliance on self-report measures raises concerns about shared method variance, which may inflate correlations between the constructs. Furthermore, our cross-sectional design does not permit causal inference. Establishing causal direction would require longitudinal or experimental designs—for example, multi-wave latent transition analyses or intervention studies that track whether changes in key predictors precede shifts in profile membership. The identified patterns can therefore only be interpreted through previous theories and findings, and the new hypotheses formulated above have yet to be followed up empirically.

Collapsing theoretically distinct threat constructs may limit the ability to determine which specific type of threat participants are perceiving. However, this approach still allows for the identification of broader patterns of outgroup threat perception, providing meaningful insights for intervention design and a clear basis for future research.

The strength of the person-centered method is also its limitation, as it can provide new insights into variable combinations we have not yet considered, although it cannot offer any information on the nature of their effects on each other. Furthermore, our findings and proposed interventions are sample specific and cannot be generalized to other receiving societies or contexts. To ensure that the profiles found in this study are stable, we recommend investigating the replicability of the results in subsequent studies using independent samples. 

## Conclusion

The results of our study show that, for most Germans, positive attitudes toward refugees and a general knowledge about their emotional struggles prevail. Conversely, Germans’ struggles with the arrival of refugees in Germany seem to lie mainly in threat perceptions and anxious emotional responses. These results highlight the benefit of taking a closer look at different intergroup emotions, as their significance for members of the receiving society can be differentiated. In each of the identified profiles, one or two emotions clearly stand out. It also becomes clearer that positive intercultural contact experiences alone cannot replace contact with actual refugees.

In general, intercultural contact should be encouraged, as it tends to have a positive effect on threat perceptions and emotional appraisals, as well as on attitudes toward refugees. However, depending on the respective profile and its characteristics of threat perceptions and negative intergroup emotions, intercultural contact experiences should be preceded by or combined with other interventions to cope with feelings of strangerhood and uncertainty. Regarding future directions for research, it would be relevant to focus on identifying factors that can lower intergroup anxiety toward refugees and increase positive intergroup emotions between members of the receiving society and refugees.

In our study, we highlighted the complexity of intergroup emotions in combination with different levels of threat perceptions as well as prior experiences and levels of cognitive empathy, accompanied by differing but mostly positive attitudes toward refugees. Practical interventions should consider this complexity and include multifaceted approaches to support all members of the receiving society in the process of forming an inclusive community that can recognize opportunities and seize them together.

## Supplementary Information

Supplementary Material 1.

## Data Availability

The dataset generated and analyzed in the current study are available in the zenodo repository, https://doi.org/10.5281/zenodo.8355252.3 [9] An MPlus Output for Latent Profile Analysis with a four-profile-solution in html-format is available in the zenodo repository as well, https://doi.org/10.5281/zenodo.17907588 [61].

## Appendix

**Table 5:** Fit indices of the two CFAs

Model	χ^2^	df	CFI/TLI	RMSEA (CI)	SRMR
1	1802.536**	644	0.947/0.936	0.044 (0.042-0.047)	0.040
2	1578.086**	583	0.951/0.940	0.043 *(0.041-0.046)*	0.041

*Note.* ***p*<.001 (two-tailed)

## Footnotes

1 Affective valence is defined as how positive or negative an individual evaluates their emotional state [67].

2 Data collection was part of a larger project. As we aimed to concentrate on Germans’ patterns of cognitive-affective-attitudinal responses toward refugees, we only included selected parts of the collected data.

3 The questions were: “Do you have German citizenship: yes/no”; “Were you born in Germany: yes/no”, “Were both your parents born in Germany: yes/no”.

4 Participants reported overall little to no contact frequency with refugees but moderate contact frequency with foreigners (Contact quantity was assessed with a single item asking how often participants interacted with foreigners in Germany, rated on a four-point scale from [1] (Almost never) to [4] (Very often) (M = 2.59, SD = 0.947) [13].

## Abbreviations

AIC: Akaike Information Criteria
ANOVA: Analysis of variance
BSSS: Berlin Social Support Scales
BIC: Bayesian Information Criteria
CFA: Confirmatory Factor Analysis
CFI: Comparative Fit Index
GESIS: Gesellschaft Sozialwissenschaftlicher Infrastruktureinrichtungen
IBM: International Business Machines Corporation
ISS: Intercultural Sensitivity Scale
ITT: Integrated Threat Theory
LL: Log-likelihood
LPA: Latent Profile Analysis
LRT: Likelihood-Ratio-Test
MLR: Maximum Likelihood Parameter
pbLRT: Parametric bootstrap likelihood ratio test
RGCT: Realistic group conflict theory
RMSEA: Root Mean Square Error of Approximation
SRMR: Standardized Root Mean Square Residuals
ssaBIC: Sample-Size-Adjusted BIC
SVR: Sachverständigenrat
UNHCR: United Nations High Commissioner for Refugees
VLMRT: Vuong-Lo-Mendell-Rubin-Test
