# Supplementary material for: A latent profile analysis of Germans’ threat perceptions, intergroup emotions and attitudes toward refugees
Source: BMC Psychol. 2026 May 20;14:780. doi: 10.1186/s40359-026-04554-x (PMC13200436; doi:10.1186/s40359-026-04554-x)
Supplement: Supplementary file 1 — Supplementary Material 1. [file 40359_2026_4554_MOESM1_ESM.docx]

**Skalen zur Erfassung psychologischer Reaktionen von Deutschen gegenüber Geflüchteten**

**(1) Demographie**

| Geschlecht: 0 Männlich 0 Weiblich |
| --- |
| Alter: ________ Jahre |
| Ist Deutsch Ihre Muttersprache? 0 Ja 0 Nein |
| Sind Sie in Deutschland aufgewachsen? 0 Ja 0 Nein |
| Wurden beide ihre Elternteile in Deutschland geboren? 0 Ja 0 Nein |
| Höchster Schul-/Hochschulabschluss:  keinen Schulabschluss  Hauptschulabschluss/ Polytechnische Oberschule nach 8.Klasse  Realschulabschluss/10. Klasse der polytechnischen Oberschule  Abitur/Fachabitur/12. Klasse polytechnische Oberschule  Abgeschlossenes Studium Uni/FH] |
| Wie hoch ist das durchschnittliche Nettoeinkommen in Ihrem Haushalt? __________ |

**(5) Symbolisches und realistisches Bedrohungsempfinden**

Reference: Allbus and Mitte-Studies; Alba, R., & Johnson, M. (2000). Zur Messung aktueller Einstellungsmuster gegenüber Ausländern in Deutschland. *Deutsche und Ausländer: Freunde, Fremde oder Feinde*, 229-253.

**1 = trifft nicht zu - 4 = trifft genau zu**

| Zusätzlich zu den in Deutschland lebenden Ausländern …] |
| --- |
| ... sind die Flüchtlinge eine Belastung für das soziale Netz. |
| ... sind die Flüchtlinge eine Bereicherung für die Kultur in Deutschland. |
| … führt die Anwesenheit der Flüchtlinge zu Problemen auf dem Wohnungsmarkt. |
| ... nehmen die Flüchtlinge den Deutschen Arbeitsplätze weg. |
| ... begehen die Flüchtlinge häufiger Straftaten als Deutsche. |
| Die Bundesrepublik ist durch die vielen Flüchtlinge in einem gefährlichen Maß überfremdet. |

**(7) Einstellungen gegenüber Geflüchteten und ihren Rechten**

Manzoni, P. (2007). Monitoring über Fremdenfeindlichkeit, rechtsextreme Orientierungen und Gewaltbereitschaft in der Schweiz. Machbarkeitsstudie. Bern: Fachstelle für Rassismusbekämpfung, Generalsekretariat des Eidgenössischen Departements des Innern.

**1 = trifft nicht zu - 4 = trifft genau zu**

| Flüchtlinge mit einer Aufenthaltsbewilligung sollten das Recht haben, enge Familienangehörige nach Deutschland nachkommen zu lassen. |
| --- |
| Flüchtlinge mit einer Aufenthaltsbewilligung sollte die Einbürgerung leichtgemacht werden. |
| Es sollte immer von der Prüfung der persönlichen Umstände abhängig gemacht werden, ob Flüchtlinge in Deutschland bleiben dürfen. |
| Das Recht auf Asyl in Deutschland sollte leichter in Anspruch genommen werden können. |

**(9) Positive interkulturelle Kontakterfahrungen**

Reference: Intercultural Sensitivity Scale (ISS), Subscale Interaction Enjoyment; Fritz & Möllenberg, 1999

**1 = trifft nicht zu - 4 = trifft genau zu**

| Ich gehe gerne mit Menschen aus anderen Kulturen um. |
| --- |
| Ich bin Menschen aus anderen Kulturen gegenüber aufgeschlossen. |
| Ich schätze die Unterschiede zwischen meinen Gesprächspartnern aus einer anderen Kultur und mir. |
| Erst wenn ich mir einen Eindruck von Menschen aus anderen Kulturen gemacht habe, nehme ich Kontakt mit ihnen auf |

**(10) Soziale Identität als Deutsche*r**

Reference: Maehler, D. B. (2012). Akkulturation und Identifikation bei eingebürgerten Migranten in Deutschland. Münster: Waxmann.

**1 = trifft nicht zu - 6 = trifft genau zu**

| Ich bin sehr stolz auf die deutsche Kultur. |
| --- |
| Ich fühle mich wohl in der deutschen Kultur. |
| Ich fühle mich eng verbunden mit der deutschen Kultur. |
| Ich bin stolz darauf, mich mit Deutschland zu identifizieren. |
| Ich fühle mich Deutsch. |

**(11) Quantitative Kontakterfahrung**

Velasco González, K., Verkuyten, M., Weesie, J., & Poppe, E. (2008). Prejudice towards Muslims in The Netherlands: Testing integrated threat theory. British Journal of Social Psychology, 47(4), 667. DOI: 10.1348/014466608X284443)

| Wie oft haben Sie in Ihrem Alltag mit in Deutschland lebenden Ausländern zu tun? [rating scale: 1= Fast nie; 2 = Manchmal, 3= Oft; 4 = sehr häufig] |
| --- |
| Wie oft haben Sie in Ihrem Alltag mit in Deutschland lebenden Flüchtlingen zu tun? [rating scale: 1= Fast nie; 2 = Manchmal, 3= Oft; 4 = sehr häufig] |

**(12) Stereotypisierung (Geflüchtete als übergriffige Männer)**

Reference:

Collani, G. & Werner, R. (2014). Ambivalent - sexistische Einstellungen gegenüber Männern (ASEM) In D. Danner & A. Glöckner-Rist (Eds.), Zusammenstellung sozialwissenschaftlicher Items und Skalen. doi: 10.6102/zis215

Glick, P., & Fiske, S. T. (1999). The Ambivalence toward Men Inventory. Psychology of Women Quarterly, 23, 519-536.

Glick, P., & Fiske, S. T. (2001). Ambivalent Sexism. Advances in Experimental Social Psychology, 33, 115-188.

**1 = trifft nicht zu - 4 = trifft genau zu**

|  |
| --- |
| Die meisten Flüchtlinge belästigen Frauen sexuell, wenn auch nur in subtiler Weise, sobald sie sich in einer Machtposition ihnen gegenüber befinden. |
| Ein Flüchtling, der sich von einer Frau sexuell angezogen fühlt, hat meist keine Skrupel, alles dafür zu tun, um sie ins Bett zu kriegen. |
| Wenn Flüchtlinge Frauen "helfen", dann tun sie das nur, um zu beweisen, dass sie Frauen überlegen sind. |
| Normalerweise versuchen Flüchtlinge, Gespräche mit Frauen zu dominieren. |

**(13b) Kognitive Empathie (Wahrnehmung von Unterstützungsbedarfen)**

Reference: Schwarzer, R. & Schulz, U. (2013) . Berlin Social Support Scales (BSSS) . Measurement Instrument Database for the Social Science. Retrieved from [www.midss.ie](http://www.midss.ie)

**1 = trifft nicht zu - 4 = trifft genau zu**

| Bitte schätzen Sie anhand der folgenden Aussagen ein, in welchem Ausmaß Flüchtlinge Unterstützung bei der Bewältigung des Alltags benötigen. |
| --- |
| Wenn Flüchtlinge niedergeschlagen sind, dann brauchen sie jemanden, der sie wieder aufbaut. |
| Für Flüchtlinge ist es wichtig, dass jemand da ist, der Ihnen zuhört. |
| Flüchtlinge brauchen jemanden, der sich um die Angelegenheiten kümmert, die sie nicht alleine erledigen können. |

**(17) Emotional reactions with regard to the refugees coming to Germany**

Reference: Folkman & Lazarus, 1985; Carver & Scheier, 1994; Ringeisen & Buchwald, 2010; Kauff M, Asbrock F, et al., 2017

**1 = Gar nicht to 5 = Äußerst**

| Bitte geben Sie an, in welchem Ausmaß die folgenden Beschreibungen auf Sie zutreffen, wenn Sie an die Flüchtlinge denken, die kürzlich nach Deutschland gekommen sind. |
| --- |
| Zuversichtlich |
| Ängstlich |
| Hoffnungsvoll |
| Sorgenvoll |
| Schuldbewusst |
| Erleichtert |
| Aufgeregt |
| Enttäuscht |
| Zufrieden |
| Beunruhigt |
| Glücklich |
| Ärgerlich |
| Furchtsam |
| Erwartungsvoll |
| Besorgt |
